# Distal radius fracture malunion in an adolescent patient treated with osteotomy and autologous iliac bone grafting: A case report

**DOI:** 10.1097/MD.0000000000022535

**Published:** 2020-10-02

**Authors:** Kai Liu, Lin Mu, Jianfeng Liu, Zhuo Fu, Lei Chen, Bin Liu

**Affiliations:** a Department of Hand and Foot Surgery, The First Hospital of Jilin University; b Jilin Province Key Laboratory on Tissue Repair, Reconstruction and Regeneration, Changchun; c Department of Radiology, The First Hospital of Jilin University, Jilin, China.

**Keywords:** autologous iliac bone grafting, distal radius fractures, malunion, osteotomy

## Abstract

**Rationale:**

Adolescent wrist trauma can cause epiphyseal dysplasia and even distal radius deformity malunion. At present, there is no uniform treatment standard for the malunion of the distal radius of adolescents. Osteotomy and autologous bone grafting are currently one of the effective ways to treat the disease. We treated an adolescent patient with distal radius deformity malunion, and used this surgical method to treat the patient and achieved satisfactory results.

**Patient concerns:**

A 16-year-old boy suffered from a serious distal radius deformity after trauma of the left wrist 8 years ago.

**Diagnoses:**

Physical examination, X-rays examination, high-resolution computed tomography scan, and 3-dimensional reconstruction images of the affected limb helped us diagnose the distal radius fracture malunion.

**Interventions:**

The fracture malunion was treated by osteotomy and autologous iliac bone grafting.

**Outcomes:**

At the 2-year follow-up, wrist flexion returned to 68°, wrist dorsiflexion to 55°, radial deviation to 14°, ulnar deviation to 12°, forearm pronation to 75°, supination to 67°. Grip strength increased to 35.1 kg after 2 years of operation, recovered to 87% of the uninjured side. Quick DASH score at 2-year follow-up was 9. No complication, such as nonunion or infection, was observed.

**Lessons:**

This rare case provides valuable insights for hand surgeons. High-resolution computed tomography scan and 3-dimensional reconstruction can help us effectively diagnose wrist diseases. Small lesions on the articular surface of the distal radius will change the position and function of the wrist joint, and cause traumatic arthritis of the wrist joint. Therefore, it is very important to reconstruct the normal structure of the distal radius articular surface. Osteotomy and autologous iliac bone grafting are effective treatments for serious distal radius fracture malunion in the adolescent patient. During the operation, care should be taken to protect the osteoepiphysis to avoid bone dysplasia.

## 1 Introduction

The incidence of distal radius fractures is approximately 0.26%.^[1]^ Malunion is a common complication of distal radius fractures treatment. The morbidity is estimated at 25% after conservative treatment and 10% after surgical treatment approximately.^[2]^ Patients with malunion of distal radius can suffer from a variety of clinical symptoms, such as posttraumatic arthritis,^[3]^ nerve neuropathy,^[4]^ cosmetic deformity, and poor functional outcomes.^[5]^ In some circumstances, therapeutic methods should be directed at symptoms rather than results of imaging examination.^[6]^ Some studies reported that the bone loss caused by osteotomy operation must be padded by cartilage,^[7]^ bone,^[8]^ or bone substitute.^[1]^ Here, we report a rare clinical case of an adolescent boy with malunion of distal radius fracture, using autogenous bone graft transfer from the ilium to the distal radius after osteotomy to recover the radiocarpal joint.

## 2 Case report

**Figure 1:**
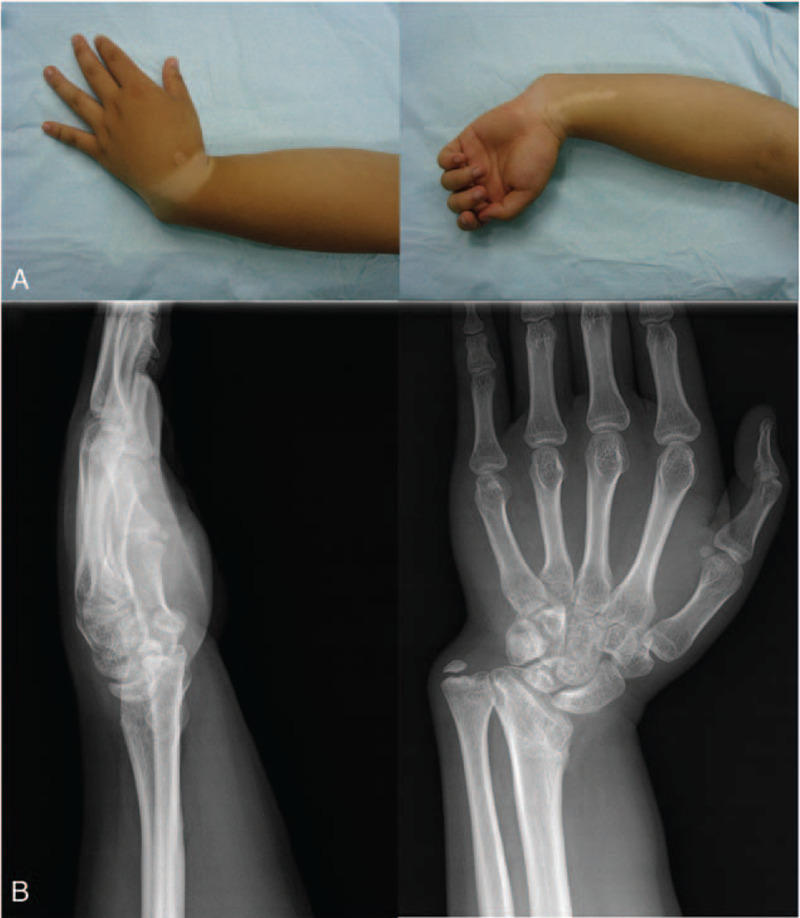
(A) Clinical pictures of serious cosmetic deformity of the left wrist. (B) The preoperative radiographs show deformity of the distal radius in both coronal and sagittal planes. Tilt of the radiocarpal articular surface and anatomical change of the carpal bones in the left wrist are shown in the images.

**Figure 2:**
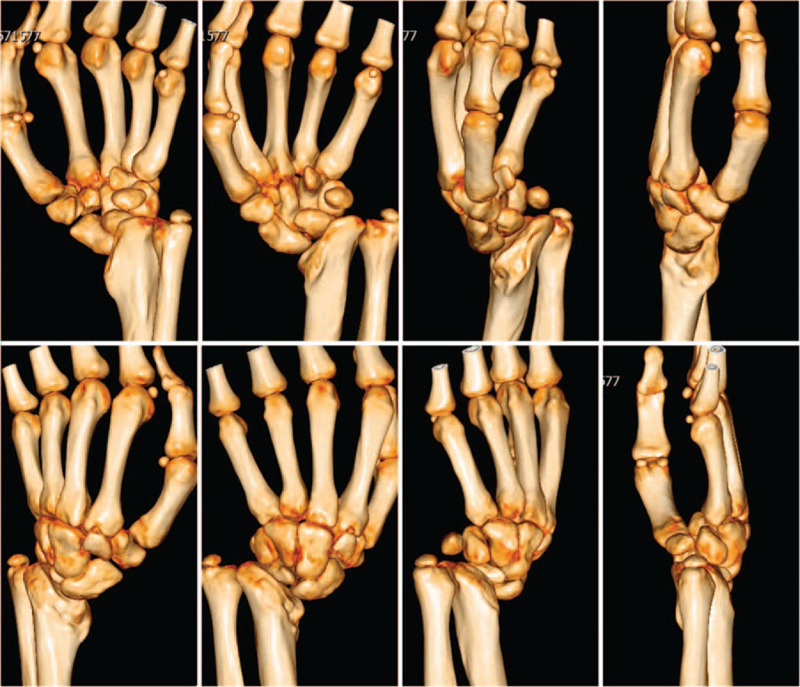
Three-dimensional reconstruction images of the left wrist.

A 16-year-old boy has suffered from serious distal radius deformity for 8 years. Primary treatment was done at the regional hospital after trauma of the left wrist 8 years ago. Thereafter, malunion of the left wrist has developed gradually. The left wrist showed severe deformity with a longitudinal scar in the middle of the wrist (Fig. 1A). At the time of physical examination, pronation and supination of the left forearm were restricted to 60° and 65°, respectively. Wrist flexion and dorsal extension were limited to 50° and 35°, respectively. Radial and ulnar deviation of the left wrist joint were restricted to 50° and negative 20°, respectively. His grip strength was assessed 3 times using the dynamometer (Sammsons Preston Ryolan, Bolingbook, IL). The mean grip strength of the affected limb was 14.9 kg, with a difference of minus 63% compared with the contralateral side (40.4 kg). The function of the affected extremity was assessed by the Quick DASH (Disability of Arm Shoulder and Hand) score from 0 (normal function) to 100 (upper limb unusable).^[9]^ Preoperative score of the affected limb was 49. Our patient also had standard lateral and posteroanterior X-rays of the left wrist (Fig. 1B). The articular surface of the left radiocarpal joint was inclined on the radial side, and the anatomical position of the left wrist carpal bone was changed. Left ulnar styloid process was free. High-resolution computed tomography (CT) scan and 3-dimensional reconstruction images were used for developing the best surgical program (Fig. 2).

**Figure 3:**
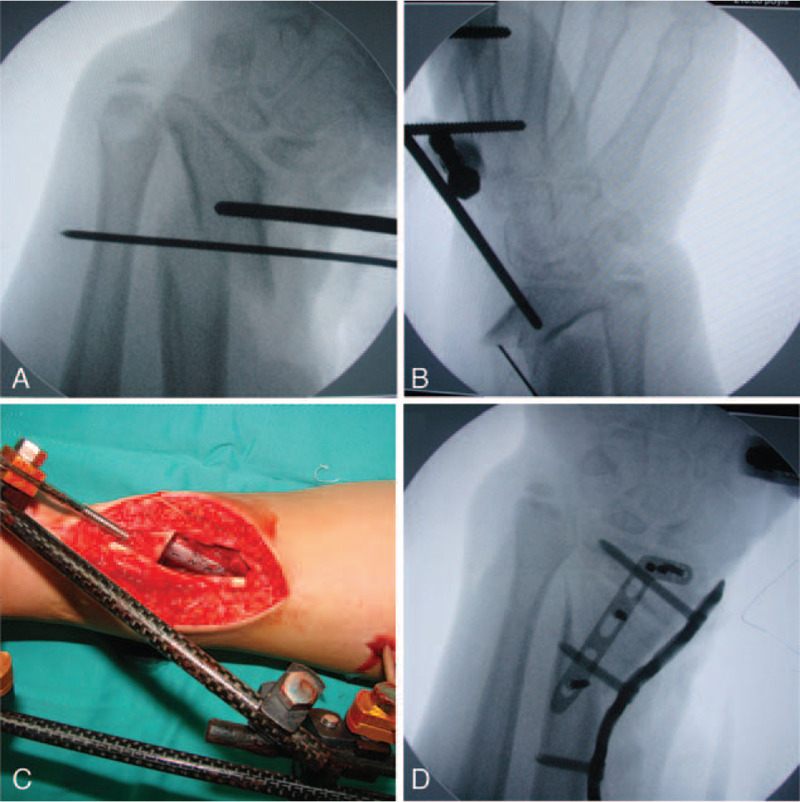
(A) A Schanz screw is employed to make the radiocarpal articular surface horizontal. (B, C) The external fixators are employed to fix the position of the Schanz screw. (D) Intraoperative radiographs confirmed the restoration of anatomy of the radiocarpal joint.

Our patient in the supine position was placed on the operating table with the left arm on a hand table in the resting position. A tourniquet was used before a 12 cm wrist radialis side longitudinal incision was made. The exposure was done with care to avoid the late osteonecrosis. The superficial terminal branch of radial nerve was carefully found and protected. Distal parts of the brachioradialis tendon and the flexor carpi radialis tendon were transected to facilitate osteotomy correction and bone graft insertion. Contracture of the soft tissues changed significantly after the contracted band tissues were removed. The extensor tendons and the sheaths were pulled aside and then the abnormal wrist joint was exposed. Intraoperative fluoroscopy images were used to assure the accurate position of osteotomy. The healthy opposite wrist was employed as the reference to design the corrective osteotomy position. Osteotomy was performed closely to the radiocarpal articular surface and ended at the proximal site of the sigmoid notch. A thin kirschner wire was drilled into the bone along the designed line as the guidance. A bone saw was used to perform the osteotomy under the guidance of kirschner wire. We maintained the integrity of the metaphyseal articular surface. Schanz screws were inserted the bone block of the distal radius to make the radiocarpal articular surface horizontal and maintained this state. Intraoperative radiographic images of the distal radius were acceptable (Fig. 3A). External fixators were used to fix positions of the screws (Fig. 3B and C). Autologous iliac crest was used to fill the bone gap at the osteotomy site. The bone gap was irregular in shape, with a volume of 3 cm × 2 cm × 1.5 cm approximately. A 3.5 mm oblique angled titanium alloy locking compression plate (Synthes GmbH, Solothurn, Switzerland) and a titanium alloy locking L-plate (Synthes GmbH, Solothurn, Switzerland) were used as a fixation device after the bone graft. Intraoperative fluoroscopy image showed the satisfactory correction results. (Fig. 3D).

**Figure 4:**
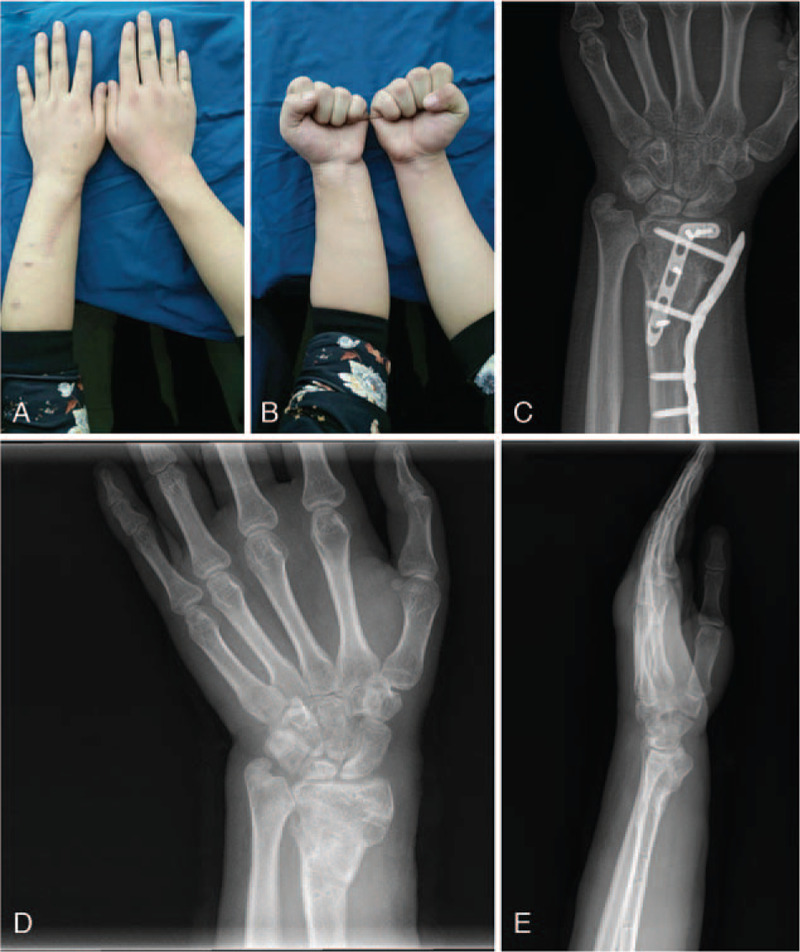
(A, B) Six months after the operation, satisfactory appearance of the wrists and (C) radiographic results of the left distal radius. (D, E) After internal fixation plates are removed, radiographic images of the wrist are gratifying.

**Figure 5:**
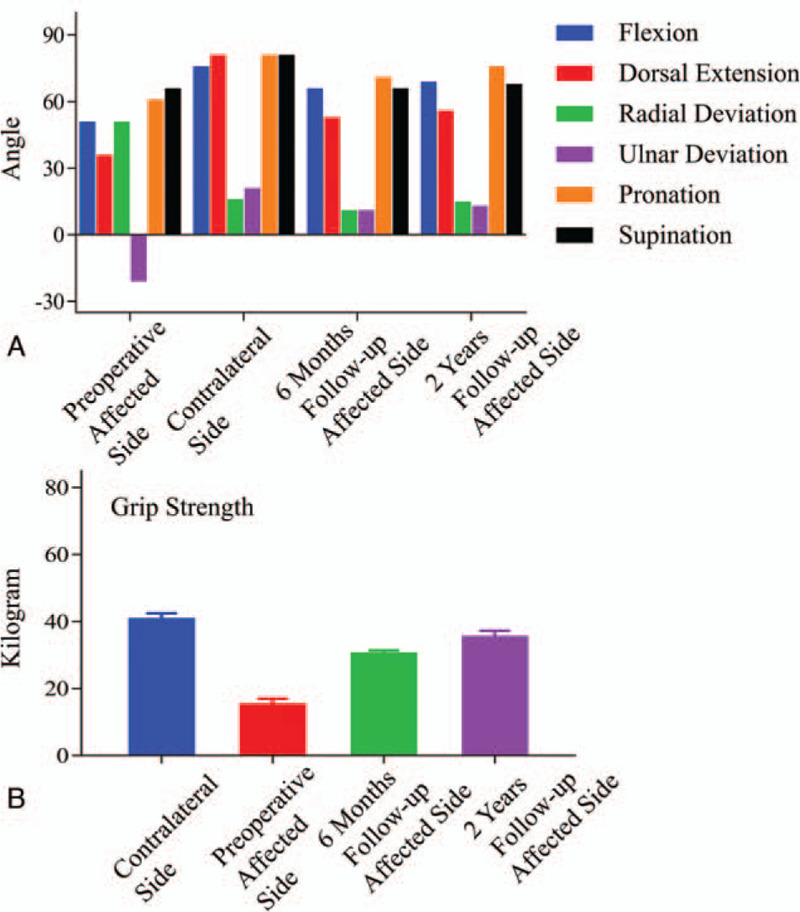
(A) Motor function recovery of the affected side at 6-month follow-up and 2-year follow-up. (B) Changes of grip strengths.

Following the internal fixation, tenorrhaphy of the severed tendons was done. A drainage tube was used before the skin closure. A plaster immobilization was provided for 6 weeks postoperatively. Six weeks after surgery, external fixators and plaster immobilization were removed, and then our patient began to try active and passive limb function exercises. At 6-month follow-up, wrist appearances and X-ray examination result were satisfactory (Fig. 4A--C). The bone graft fracture line was blurred. The position of the radiocarpal joint surface and carpal bone returned to normal. The internal fixation position was good without loosening, shifting, and breaking. Our adolescent patient also showed an improvement in wrist function. On average, the range of motion improved 15° on wrist flexion, 17° on dorsal extension, negative 40° on radial deviation of wrist joint, 30° on ulnar deviation, 10° on forearm pronation, and no change of supination. Internal fixation plates were removed at the second years after operation and fluoroscopy images were satisfactory (Fig. 4D and E). The fracture line was basically invisible, and the internal fixation holes were visible. There were no obvious abnormalities in the joint surface of the radiocarpal joint and the position of the carpal bone. At 2-year follow-up, there was no growth discrepancy between the 2 upper limbs. Wrist flexion improved 3° compared with the previous follow-up, 3° on dorsal extension, 4° on radial deviation of wrist joint, 2° on ulnar deviation, 5° on forearm pronation, and 2° on supination (Fig. 5A). Six-month postoperative grip strength (30.2 kg) increased 15.3 kg, compared with the preoperative test, with a remaining difference of minus 25% compared with the contralateral side. Grip strength increased to 35.1 kg after 2 years of operation, recovered to 87% of the uninjured side (Fig. 5B). No complication, such as nonunion or infection, was observed. Quick DASH scores at 6-month and 2-year follow-ups were 15 and 9, respectively.

## 3 Discussion

To the best of our knowledge, there was no formal classification of distal radius malunion. But some authors attempted to define the unacceptable healing of distal radius fractures as:

(1) Radial inclination < 10°
(2) Volar tilt > 20°, dorsal tilt > 20°
(3) Radial height <10 mm
(4) Ulnar variance > 2+
(5) Intra-articular step or gap >2 mm^[10]^

Apparently, the radial height of our patient was unacceptable. On the preoperative posteroanterior X-ray images, the radial styloid was under the tangent drawn at the ulnar pole. Therefore, the radial height of the left wrist of our patient must be less than 10 mm. We could clearly see a significant improvement in postoperative radiographic images of our patient, particularly the radial height and the radiocarpal articular surface location. The pathogeny could be explained by the fact that trauma of his left wrist 8 years ago damaged the osteoepiphysis of the radiocarpal articular surface. This caused dysplasia of the lateral column of radius and the styloid process of radius and changed anatomical location of the carpal bones correspondingly. Previous studies reported a systematic approach on surgical treatment of distal radial malunions (Table 1).^[11]^ The guideline of therapeutic method choice was to improve wrist motion and grip strength. Therefore, our patient was classified to the group I due to the acceptable distal radioulnar joint (DRUJ) and the unacceptable radial measurements and radioulnar length. The objective of corrective osteotomy was to recover anatomic relation of the wrist joint and the distal radius. We selectively removed the contracted soft tissue during the operation. We tried our best to preserve the supporting ligaments and microvessels while achieved the effect of osteotomy to avoid occurrence of postoperative nonunion.

**Table 1:**
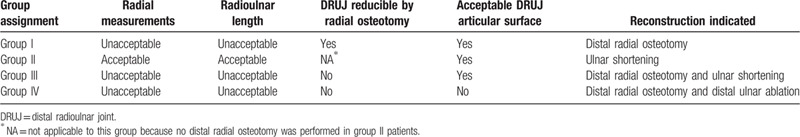
Criteria for patient grouping and treatment recommendations in study by Graham and Hastings^[11]^.

At the time of follow-up, we found the significant advancement of wrist function and grip strength. Because the preoperative DRUJ of our patient was intact, the function of pronation and supination of the affected limb was not significantly affected. The DASH score of the affected limb distinctly improved and no complication was observed. It was evident that complex malunion of the distal radius in adolescents could benefit from corrective osteotomy and bone autograft.

Grip strength of the affected extremity at 6-month follow-up increased 15.3 kg compared with the preoperative test and recovered to 75% of the uninjured side. However, some study reported that grip strength of affected limb after surgery recovered up to 80% to 85% of uninjured side.^[12]^ We considered that postoperative functional exercise could be instrumental in recovering grip strength of young patients gradually. At 2-year follow-up, grip strength of the affected extremity recovered up to 87% of uninjured side.

Clinical assessment grades of wrists (Table 2)^[13]^ consisted of description of pain, range of active motion and grip strength. On the basis of the results of these assessments, wrists were divided into 4 grades: very good, good, fair, and poor. Our patient was at the poor grade preoperatively result from constrained movement of the wrist joint and reduction of grip strength to less than 40% of the normal side. After the operation, he was classified to the good grade because of moderate limitation motion of the wrist joint and recovering of grip strength.

**Table 2:**
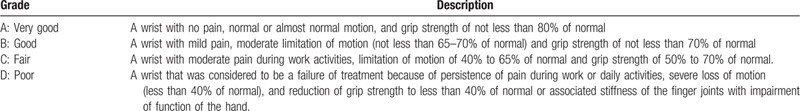
Clinical assessment of wrists with respect to pain, range of active motion, and grip strength^[13]^.

For underage patients with joint trauma, it is essential to consider the risk of progression to arthritis. If joint malunion exists in a patient below 50 years of age, the risk of arthritis will be 10 times greater than other patients, whereas it will be only 2 times greater in sufferers above 65 years of age.^[14]^ Our patient was classified to the grade 0 of grading of post-traumatic arthritis (Table 3).^[15]^ However, we must be also aware of the risk of traumatic arthritis persists even 20 years after the fracture.^[16]^

**Table 3:**
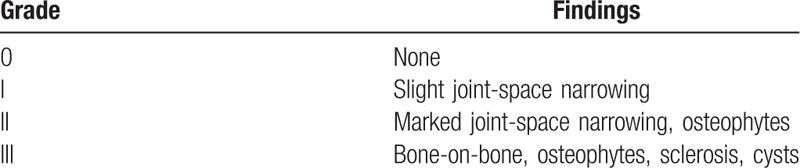
Grading of post-traumatic arthritis^[15]^.

In the present case, corrective osteotomy and bone autograft technique were used not only to correct joint malformation but also to cure cosmetic deformity and mentality of the self-contemptuous boy. This rare case demonstrates that an adolescent serious distal radius malunion must be systematic treated and operation of osteotomy and autologous iliac bone grafting can be considered. The osteoepiphysis of underage patients should be protected carefully during the operation.

## Author contributions

**Conceptualization:** Bin Liu.

**Data curation:** Jianfeng Liu.

**Formal analysis:** Zhuo Fu.

**Investigation:** Lin Mu

**Resources:** Lei Chen.

**Writing – original draft:** Kai Liu.

**Writing – review & editing:** Bin Liu.
